# Innovative Atherosclerosis Models: Advancing Pathophysiology and Translational Research

**DOI:** 10.34133/research.0617

**Published:** 2025-02-20

**Authors:** Huiting Jiang, Yukun Liao, Mengliang Zhu, Luksika Jiramonai, Hongyun Wu, Yixin Zhong, Zulong Xie, Xing-Jie Liang

**Affiliations:** ^1^ Department of Cardiology, Second Affiliated Hospital of Chongqing Medical University, Chongqing 400010, China.; ^2^ Department of Radiology, Second Affiliated Hospital of Chongqing Medical, Chongqing 400010, China.; ^3^ Department of Nuclear, Affiliated People’s Hospital of Chongqing University, Chongqing 401121, China.; ^4^ CAS Key Laboratory for Biomedical Effects of Nanomaterials and Nanosafety, CAS Center for Excellence in Nanoscience, National Center for Nanoscience and Technology of China, Beijing 100190, China.; ^5^ University of Chinese Academy of Sciences, Beijing 100049, China.

## Abstract

Atherosclerosis (AS) is a chronic inflammatory condition influenced by glucose and lipid disorders, oxidative stress, and thrombosis, reflecting the complexity of its pathological process. The development of accurate experimental models that simulate human AS is essential for understanding its initiation and progression. This review summarizes the current AS research models and analyzes their specific application scenarios. We discuss tissue-engineered blood vessels (TEBVs) and vessels-on-a-chip (VoCs), which leverage tissue engineering and precise microenvironmental control to construct in vitro models that closely resemble the structure and function of human AS. Isolated vessel segments from live animals provide a valuable tool for investigating human AS due to their physiological similarity, controllability, and reproducibility. The review further outlines the construction of AS animal models through high-fat diets and gene-editing techniques, highlighting how immune-inflammatory responses, mechanical arterial injury, and hemodynamic changes accelerate model development. This comprehensive analysis highlights the potential of AS models to revolutionize theranostic applications in clinical translational research, paving the way for more personalized and effective treatments for AS in the near future.

## Introduction

Atherosclerosis (AS) is the primary pathological foundation of ischemic cardiovascular diseases, including myocardial infarction, peripheral arterial disease, and stroke. Despite marked advances in managing conventional risk factors, a high residual risk of AS persists due to factors such as obesity, sedentary lifestyle, and unhealthy dietary patterns [1]. Therefore, exploring the underlying mechanisms of AS and developing novel therapeutic strategies are critical for effective clinical management. To facilitate successful clinical translation of basic research, it is essential to employ tailored experimental models—whether in vitro, ex vivo, or in vivo—designed to align with the specific objectives of each study.

**Fig. 1.**
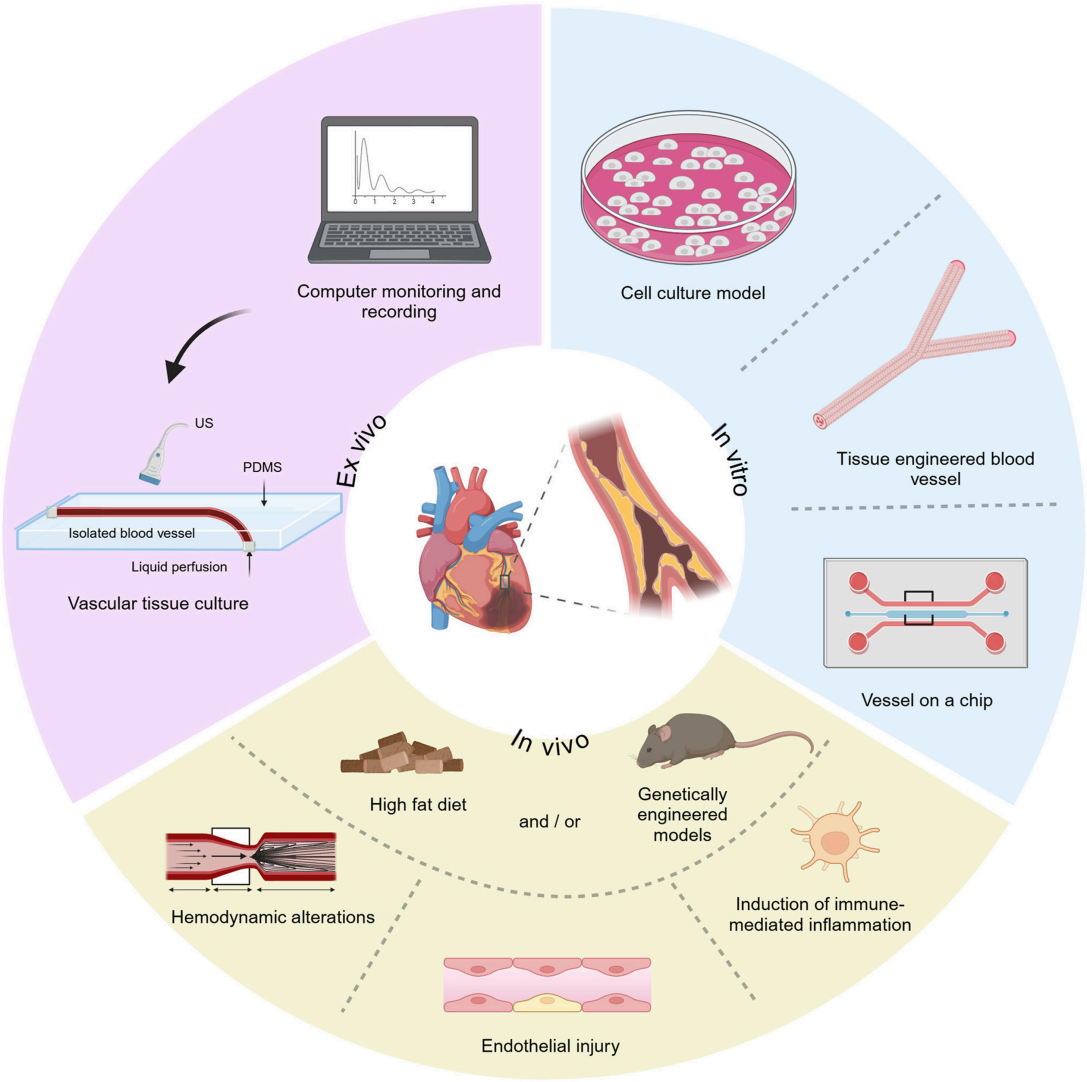
A schematic overview of the main experimental models used in AS research is presented. These models can be categorized into 3 major groups: in vitro systems (including 2D and 3D AS models), ex vivo systems, and animal models used for translational medical research relevant to humans. In vitro models include single-cell type culture systems as 2D models and more complex 3D models, such as TEBVs and VoC platforms. TEBVs serve as alternatives to natural blood vessels for personalized diagnosis and treatment. The VoC system replicates physiological conditions in vitro and is designed to mimic the function and microenvironment of human blood vessels. Ex vivo models use isolated blood vessels cultured in bioreactor systems, providing a controlled, physiologically relevant environment for studying the roles of AS-related cellular interactions and hemodynamics in the pathogenesis of the disease. In vivo, atherosclerotic plaques are induced by high-fat diets alone or in combination with genetic modification in mice. Plaque formation is further accelerated by immune-mediated inflammatory responses, vascular endothelial injury, and altered hemodynamics. By employing a combination of these experimental models, researchers can achieve a comprehensive understanding of AS, thereby facilitating the development of targeted therapies and personalized treatment strategies.

In early studies, research focused primarily on the single-cell type models and genetically modified mice [2–4]. However, single-cell type models are limited in their ability to replicate the complex in vivo microenvironment of atherosclerotic plaques, neglecting the interactions and mutual regulation between various cell types. Additionally, animal models take considerable time to develop, and lesions often form in the aorta rather than in human-specific sites like the coronary and cerebral arteries [5]. The inability of mice to form vulnerable plaques hinders their ability to accurately represent the characteristics of human plaques that are prone to rupture and trigger clinical events [6,7]. In recent years, advances in nanomaterials, molecular imaging, and other technologies have importantly improved AS modeling. Among these innovations, 3-dimensional (3D) models have gained attention for their economic and ethical advantages, as well as their ability to isolate and analyze numerous contributing factors in this complex disease. In in vivo models, the disease modeling process can be accelerated by inducing immune-inflammatory responses, vascular endothelial damage, and hemodynamic changes. Specific disease features can be accentuated by modifying dietary regimens or utilizing gene-editing technology. These approaches allow models to better mimic the characteristics of human plaques and reduce the time required for model development. This review highlights the evolution of AS models, tracing the transition from 2D to 3D systems, while incorporating various perspectives on the pathogenesis of AS (Fig. 1).

## A Brief Overview of AS

**Fig. 2.**
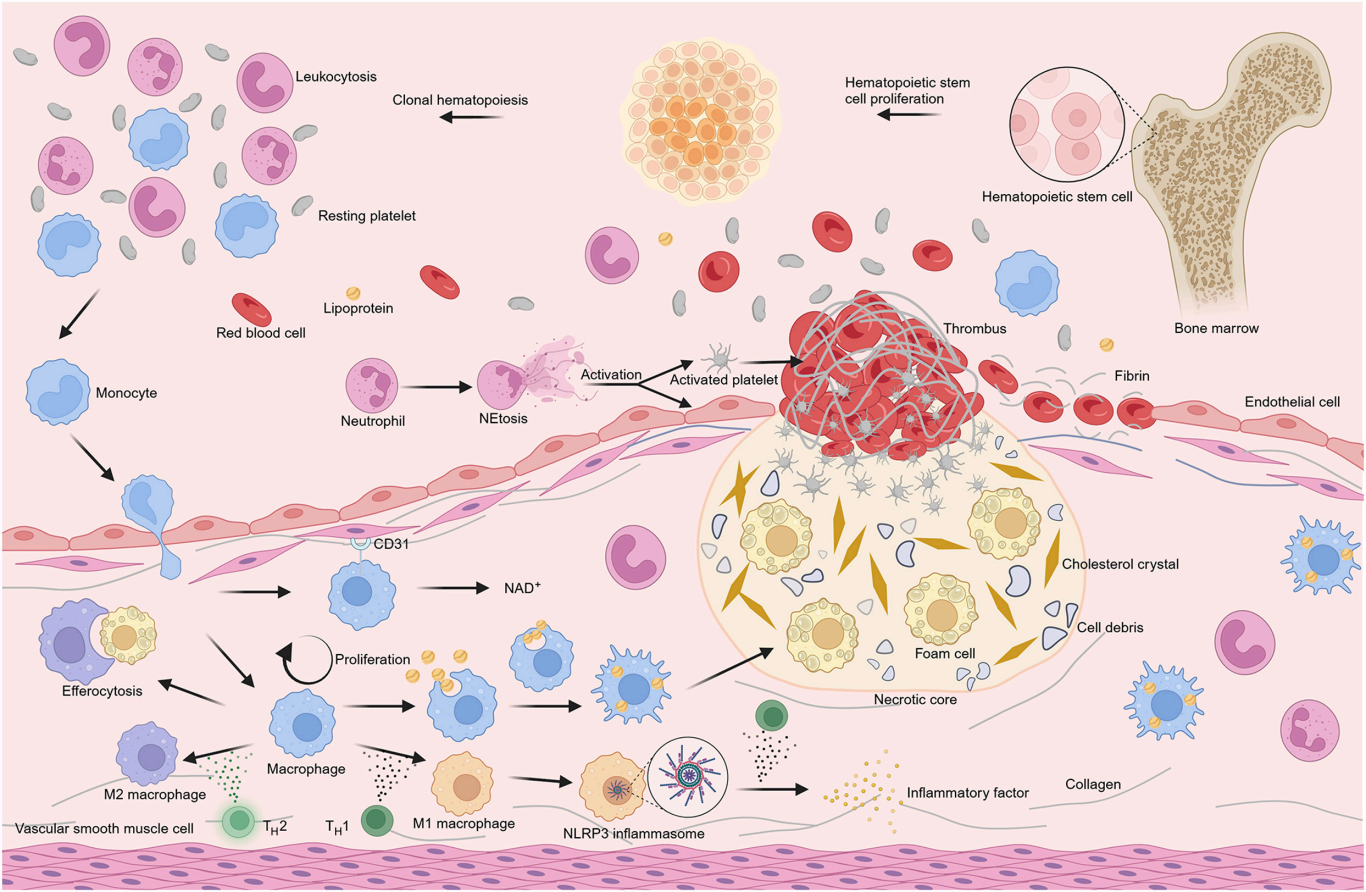
Monocytes are chemotactically recruited to the intima, where they differentiate into macrophages. These macrophages engulf lipids and become foam cells. They can polarize into proinflammatory M1 phenotype, exacerbating inflammation by releasing cytokines and promoting T cell activation. Alternatively, they can polarize into reparative M2 phenotype to resolve excessive inflammation through efferocytosis and resolution. Accumulation of foam cells, cell debris, and cholesterol forms the necrotic core of the plaque, triggering further inflammation and recruitment of neutrophils, monocytes, and other inflammatory cells. CHIP leads to leukocytosis and emergence of clones with pro-inflammatory properties, enhancing plaque instability through NLRP3 inflammasome and NETs. Plaque rupture exposes collagen and subcollagen, releasing vWF and activating platelets, ultimately causing acute thrombus formation.

AS is characterized by the formation of a lipid and necrotic core, with lesions that accumulate extracellular lipid particles, foam cells, and debris within the intima of arterial walls. Overlying this core is a fibrous cap, composed of a collagen-rich matrix and smooth muscle cells (SMCs), which is covered by a layer of dysfunctional endothelial cells (ECs). Inflammatory cells, predominantly macrophages and T lymphocytes, infiltrate the lesions, promoting plaque progression and contributing to the vulnerable plaques. The impairment of macrophage efferocytosis, responsible for the clearance of apoptotic cells within plaques, has been implicated in the growth of necrotic cores and exacerbation of inflammation [8]. When a vulnerable plaque ruptures, it can trigger thrombosis, leading to clinical ischemic events (Fig. 2) [9–11]. Since the first report of AS in 1904, various hypotheses have been proposed, including the modified response-to-injury hypothesis, oxidative modification hypothesis, hemodynamic theory, and inflammatory hypothesis [12], to elucidate its complex etiology. Clonal hematopoiesis of indeterminate potential (CHIP), along with the consequent elevation in circulating leukocytes, including monocytes and neutrophils, has recently emerged as a novel contributor to AS. This is partially mediated through the activation of the NLRP3 inflammasome and the formation of neutrophil extracellular traps (NETs) [13,14]. These theories have guided the development of experimental models, although many of these models tend to focus on specific aspects of the disease to simplify analysis. As a result, new experimental models are essential for accurately replicating the diverse and complex pathological processes of AS, providing a more precise simulation of disease mechanisms.

Current management of AS primarily focuses on prevention and lifestyle modifications. Lipid-lowering drugs are the standard pharmacological therapy for improving dyslipidemia and stabilizing plaques [15]. Antiplatelet therapy or a combination with anticoagulation therapy can be selected based on the risk of thrombosis. In cases where thrombosis or plaque-induced vascular stenosis occurs, common surgical interventions include balloon angioplasty, stent implantation, endarterectomy, and bypass grafting. Although these treatments have improved quality of life, challenges remain in reversing plaque progression, reducing restenosis rates after endovascular surgery, and minimizing surgical trauma [16,17]. Emerging therapeutic strategies, such as nanomedicine, immunotherapy, and gene modification therapy, offer the potential to further enhance treatment efficacy [18]. As these novel approaches continue to develop, experimental models will serve as pivotal tools in validating therapeutic efficacy and providing indispensable platforms for translational medical research.

## In Vitro AS Models

Since the development of AS involves ECs, SMCs, macrophages, and lymphocytes, in vitro experiments are an important component of preclinical research. Based on the cell culture platform and its interaction with the extracellular matrix, in vitro models can be classified into 2D and 3D models. Advances in biomanufacturing technologies have made it possible to develop precise in vitro models for a deeper understanding of the pathophysiology of AS.

### 2D AS models

#### Single-cell type models

Single-cell type culture systems involve the growth of cells on flat plastic dishes, where they adhere and proliferate. This system allows for the study of cell behavior using cost-effective chaperones. The methods and applications of cell culture are summarized in Table 1. Commonly used materials for in vitro cell studies include primary cells and immortalized cell lines. In recent years, human induced pluripotent stem cells (hiPSCs) have emerged as a transformative tool in cardiovascular research, offering the ability to differentiate into key cell types such as SMCs, ECs, and macrophages [19]. They can also be differentiated into neutrophils, natural killer (NK) cells, and neurons, enabling detailed studies of cellular interactions in AS. For example, hiPSC-derived neutrophils provide insights into inflammation and plaque formation [20], while NK cells and neurons derived from hiPSCs help elucidate cytotoxic effects and the neurological impact of AS, respectively [21]. With their capacity for self-renewal and differentiation, hiPSCs are invaluable for in vitro disease modeling, personalized medicine, and drug screening. Additionally, their ability to recapitulate patient-specific genetic and phenotypic features supports research into individual disease variability and therapeutic responses. Since hiPSCs are derived from somatic cells, they bypass ethical concerns associated with embryonic stem cells and reduce the risk of immune rejection in regenerative therapies. These qualities underscore their immense potential in cardiovascular research. However, 2D cell cultures have substantial limitations. The planar nature of the 2D system makes it impossible to accurately replicate the real conditions of cell growth and diffusion. Additionally, it cannot simulate the native structure of blood vessels and the characteristics of human plaques. Therefore, directly translating drug trial results obtained in a laboratory setting to patients carries potential risks.

**Table 1.** Common cell stimuli in AS experimental models. TNF-α, tumor necrosis factor-α; IL-1β, interleukin-1β; IFN-β, interferon-β; CXCL1, chemokine (C-X-C motif) ligand 1; LTB4, leukotriene B4; PAF, platelet activating factor; LPC, lysophosphatidylcholine; LPS, lipopolysaccharide; oxLDL, oxidized low-density lipoprotein; Ang II, angiotensin II; AGEs, advanced glycation end products; Cp, *C. pneumoniae*.

Model	Stimulus	Reference dose	Focus	References
Chemical stimuli	Cytokines	50 ng/ml TNF-α, 24 h	SMC apoptosis	[96]
		0.1 ng/ml IL-1β , 12 h	ROS production	[97]
		300 U/ml IFN-γ, 24 h	Macrophage activation	[98]
	Chemokines	20 nM CXCL1, 2 h	Neutrophil adhesion	[99]
	Leukotrienes	1 μM LTB4, 21 h	SMC migration	[100]
	Histamine	10 μM histamine, 20 h	EC activation and monocyte adhesion	[101]
	C5a	3 nM C5a, 1 h	Neutrophil activation	[102]
Pathological stimuli	LPS	1 μg/ml LPS , 6 h	EC activation and macrophage activation	[103]
Oxidative stress stimuli	H_2_O_2_	100 μM H_2_O_2_, 24 h	SMC apoptosis	[104]
Internal environment disturbance stimuli	oxLDL	40 μg/ml oxLDL, 24 h	Macrophage inflammation	[105]
	Cholesterol crystals	1 mg/ml, 24 h	Neuroinflammation	[106]
	Ang II	0.2 μg/ml Ang II, 1 h	SMC proliferation and migration	[107]
	AGEs	200 μg/ml AGEs, 24 h	Macrophage polarization	[108]
Toxic stimuli	*Cp*	4 IFU/cell Cp, 4 h	EC and SMC proliferation	[109]
	*P. gingivalis*	*P. gingivalis* [multiplicity of infection (MOI) 1:100], 2 h	EC oxidative stress and inflammatory response	[110]

#### Coculture models

Coculture models in AS research are designed to emulate the multifaceted cellular environment of the vessel wall, where the disease initially forms. These models recognize the complex interactions between various cell types, including neutrophils, NK cells, and neurons, which are increasingly recognized for their roles in atherosclerotic pathology. For instance, the coculture of ECs and neural cells in vascularized organoid models provides a powerful platform to investigate the crosstalk between the brain and vasculature, shedding light on the signaling pathways driving vascular inflammation in AS. To better mimic in vivo conditions, coculture systems often involve combining different cell types at specific ratios, facilitating the study of both direct and indirect cell–cell interactions [22,23]. Indirect coculture models, which focus on cell-dependent interactions without physical contact, are also employed to investigate paracrine signaling and soluble factor exchanges. Researchers use conditioned media (CM) [24], molecular materials [25], porous membranes [26], transwell systems [27], or flow chambers [28] to create these environments. Although 2D coculture systems exposed to culture media offer an effective platform for AS research and development, especially for studying intercellular signal transduction, these models do not accurately represent the spatial characteristics of atherosclerotic plaques.

### 3D AS models

A profound understanding of the spatial organization of plaque structure and the relationship between individual cellular components and plaque stability is important for designing effective treatment protocol and promote drug discovery. Consequently, researchers have employed in vitro 3D models for AS studies, which outline the 3D structure of the organ, incorporated with a naturally growing extracellular matrices (ECM). Computational models and biophysical simulations have been used to enhance the interpretation of experimental data. The main 3D models include spheroid cultures [29,30], parallel plate flow chambers [31], membrane culture dishes [32], tissue-engineered blood vessels (TEBVs), and vessels-on-a-chip (VoCs). TEBVs and VoCs, which closely resemble the anatomical structure of blood vessels, can effectively simulate the effects of shear force, chemical induction, and other factors on AS. These 2 models have become the widely used in vitro culture devices, and this section will focus on their latest advancements (Table 2).

**Table 2.** TEBVs and VoCs for AS studies. AS, atherosclerosis; TEBVs, tissue-engineered blood vessels; hECFCs, endothelial colony forming cells; hNDF, human neonatal dermal fibroblasts; hCASMCs, human coronary artery smooth muscle; viSMCs, iPS-derived smooth muscle cells; viECs, iPS-derived endothelial cells; EPCs, endothelial progenitor cells; SMCs, smooth muscle cells; ECs, endothelial cells; hEPCs, human umbilical cord blood-derived endothelial progenitor cells; UASMCs, umbilical artery smooth muscle cells; VoCs, vessels-on-a-chip; HUVECs, human umbilical vein endothelial cells; HTPs, heated tobacco products.

Model	Technique	ECM material	Cell sources	Outcomes	References
TEBV	Mold-casting	Collagen I	Primary hECFCs, primary hNDF, hCASMCs, primary human monocytes, and U937	Simulation of early-stage AS	[33]
		Collagen I	viSMCs and viECs	Identified the role of the endothelium	[34]
	Decellularization	Decellularized blood vessel	EPCs and SMCs	A20 gene-transfected TEBVs had an anti-atherosclerotic effect	[36]
	Scaffold	No	ECs and SMCs	The relationship between vascular structure and AS	[37]
		Collagen I	hEPCs, UASMCs, and HL-60	Observation of the interaction between immune cells and blood vessels	[38]
VoC	Soft lithography	Collagen I	Primary ECs, primary SMCs, and THP-1	Building a multilayered platform to study the activities of SMCs	[111]
		No	HUVECs and THP-1	Studying leukocyte–endothelial interactions using a stenosis model	[41]
		Collagen I	HUVECs	Validating YAP implications in EC biomechanics	[112]
		Collagen I	HUVECs and neutrophils	Engineering stenotic vascular channels to study hemodynamics and cell interactions	[42]
		Fibronectin	HUVECs	Investigation of platelet aggregation at defined positions	[44]
		Fibronectin	HUVECs	Establishing an experimental platform for early-stage AS	[45]
		Collagen I	No	Simulation of late-stage atherosclerotic plaque rupture and platelet aggregation	[46]
	Molding	No	HUVECs	Optimization of Pt-NP-based drugs by simulating early microenvironments on chips	[47]
		Fibronectin	HUVECs	Develop a platform for analyzing restenosis	[51]
		Gelatin	HUVECs	Analyzing hemodynamics with narrow microchannels	[113]
	Sacrificial bioprinting	Collagen I	HUVECs	Developing an economical microvascular network	[52]
	3D printing	No	HUVECs	Preparation of liquid biopsy tools for cardiovascular risk stratification	[53]
	OrganoPlate	Collagen I	Primary HCAEC and THP-1	Analyzing how HTP reduces the risk of AS	[54]

#### Tissue-engineered blood vessels

Recent advancements in TEBV aim to bridge the gap between in vitro and in vivo models through techniques, including cell-sheet engineering, electrospinning, mold casting, decellularization, scaffold fabrication, and 3D bioprinting. These approaches enable the integration of individualized characteristics of AS patients such as vascular structure and flow characteristics under diseased conditions into the models. This improved TEBV technology offers greater adaptability, allowing for more precise application of stressors to specific cell types, rather than focusing solely on systemic effects.

**Fig. 3.**
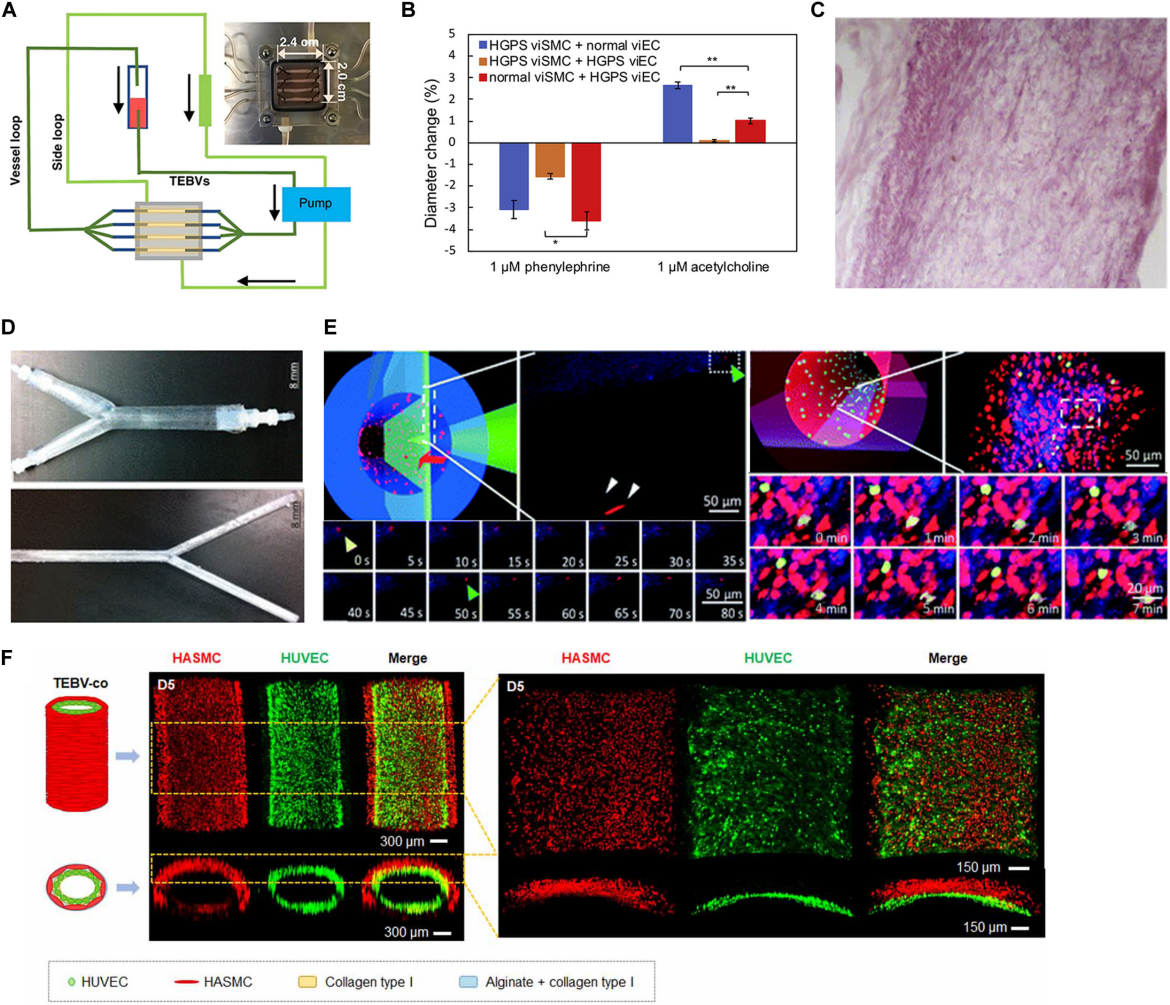
Recent advances in TEBV models for AS research. (A) Illustration of the process for preparing multilayer TEBVs using mold casting method. Copyright 2020, Nature Portfolio. (B) The TEBVs constructed from vascular endothelial cells (viECs) differentiated from induced pluripotent stem cells (iPSCs) of patients with HGPS exhibited significantly reduced responses to both phenylephrine-induced constriction and acetylcholine-induced dilation. This phenomenon was less influenced by the source of their internal SMCs (viSMCs), whether derived from normal individuals or HGPS patient-derived iPSC, compared to the source of viECs. Copyright 2020, Cell Press. (C) Hematoxylin and eosin (H&E) staining results demonstrates the morphology of the vascular matrix after decellularization, ensuring that endogenous cells had been completely removed while preserving the extracellular matrix structure of the vessel. Copyright 2008, Elsevier. (D) Geometric shapes of the common carotid artery bifurcation (upper) and the left main coronary artery bifurcation (lower) were acquired using clinical imaging techniques. Based on these, 3D models for computer simulation analysis and biocompatible stents for in vitro model studies were created. Copyright 2014, Oxford. (E) TEBVs utilize real-time imaging technology to observe the dynamic processes within the vessel, allowing researchers to monitor the perfusion, adhesion (upper), and migration (lower) of monocytes. The white arrows indicate monocytes within the TEBV lumen infusion, which are shown to be moving rapidly during imaging. The yellow arrows denote that monocytes adhered to the ECs. The green arrows represent monocytes that have traversed the EC layer from the vascular lumen into the vascular wall matrix. Copyright 2014, Royal Society of Chemistry. (F) Schematic representation of cocultured HUVECs and HASMCs within a TEBV. This illustration depicts the distinct alignment of HUVECs and HASMCs within the TEBV structure, highlighting the parallel arrangement of HUVECs and the perpendicular alignment of HASMCs. The unique arrangement is a result of the shear forces experienced during the extrusion process, which mimics the natural orientation of cells within native blood vessels. Copyright 2020, IOP Publishing.

To mimic the layered structure of natural blood vessels, Zhang et al. [33] developed arteriole-scale human TEBVs by compression molding and perfusion, incorporating primary human-derived ECs, SMCs, and fibroblasts (Fig. 3A). After endothelialization, these TEBVs were perfused with a culture medium containing oxidized low-density lipoprotein (oxLDL) and tumor necrosis factor-α (TNF-α) to establish an in vitro model of early-stage AS. These 3D vessel models accurately simulate the pathophysiological features of early-stage AS, including impaired vasodilation, increased expression of inflammatory factors/chemokines, and enhanced adhesion, migration, and differentiation of circulating monocytes. Consequently, they provide a valuable platform for evaluating the therapeutic effects of drugs targeting specific vascular functions.

There is a variable and limited proliferative potential of human primary cells, affecting the ability to generate TEBVs, as well as higher costs and longer processing times required for TEBV generation. By using ectopic expression of stem cell factors, human somatic cells can be transformed into induced human pluripotent stem cells (iPSCs). In the development of TEBVs, iPSCs ensure that the vascular endothelial cells (viECs) and smooth muscle cells (viSMCs) in the model are derived from the same donor iPSC line, thus mimicking the cellular composition and function of human blood vessels. Atchison et al. [34] applied this approach to assess the progression of atherosclerotic lesions in Huntington–Gilford progeria syndrome (HGPS) and to investigate interactions between different cell types. The TEBV model revealed greater insight into the mechanisms underlying vascular lesions in HGPS, as evidenced by a decrease in vasoconstriction and diastolic function (Fig. 3B), as well as an increase in the expression of inflammatory markers such as VCAM-1 and E-selectin. The use of hiPSCs also allows for the creation of 3D vascular structures, such as vascular rings or organoids, which can be used to study the spatial organization of cells within the vessel wall and their response to various stimuli. These 3D vascular models can be further integrated with other cell types, such as pericytes and fibroblasts, to mimic the complexity of the arterial wall.

Furthermore, in vitro ECs can adhere to decellularized scaffolds derived from heterologous blood vessels to construct TEBVs [35]. When combined with gene-editing technologies, these models hold great promise for precision medicine (Fig. 3C) [36]. Another widely accepted approach involves the use of biodegradable stents combined with cultured cells. For example, Martorell et al. [37] seeded ECs and SMCs into vascular-like structures tailored to the specific geometry of vessels with atherosclerotic plaque (Fig. 3D). This approach, along with bioreactor integration and long-term flow culture methods in AS models, enables the simultaneous assessment of antiproliferative and antithrombotic effects of treatments. Recent advancements in TEBVs also involve the use of real-time imaging technology to explore biomechanical factors in vascular function and disease progression. This in-depth investigation allows for the screening and development of new drugs. By utilizing a TEBV in a specialized perfusion and imaging chamber filled with a leukocyte-containing medium, researchers can simulate the influences of hemodynamics on the vessel wall and ECs. Deep-penetrating 2-photon laser microscopy further provides the real-time insights into the impact of blood flow shear forces on leukocyte adhesion [38]. This approach importantly enhances our understanding of immune cell interactions with the vascular endothelium (Fig. 3E). Interestingly, Bosch-Rué et al. [39] have integrated 3D bioprinting with scaffold fabrication techniques to extrude 2 distinct biomaterials—sodium alginate and collagen—along with cell mixtures comprising human umbilical vein endothelial cells (HUVECs) and human aortic smooth muscle cells (HASMCs) through a nozzle, creating a multilayered structure (Fig. 3F). During the extrusion process, cells are subjected to shear forces that can direct their alignment in specific orientations. For instance, HUVECs tend to align parallel to the TEBV under the influence of shear forces, while HASMCs show a preference for perpendicular alignment to the TEBV. This technology is advantageous for fabricating vascular mimics with both biological activity and structural stability while preserving the natural alignment and functionality of the cells. Moreover, this approach holds potential value in the fields of tissue engineering and regenerative medicine, particularly in the fabrication of vascular grafts.

#### Vessels-on-a-chip

VoCs integrate multiple disciplines, including micromachining technology, microfluidic technology, and cell biology, to simulate blood vessel stenosis and disturbed flow (d-flow) microenvironment. In general, the primary methods for fabricating the chips include soft lithography, molding, microcontact printing, laser ablation, micromachining, and 3D printing.

**Fig. 4.**
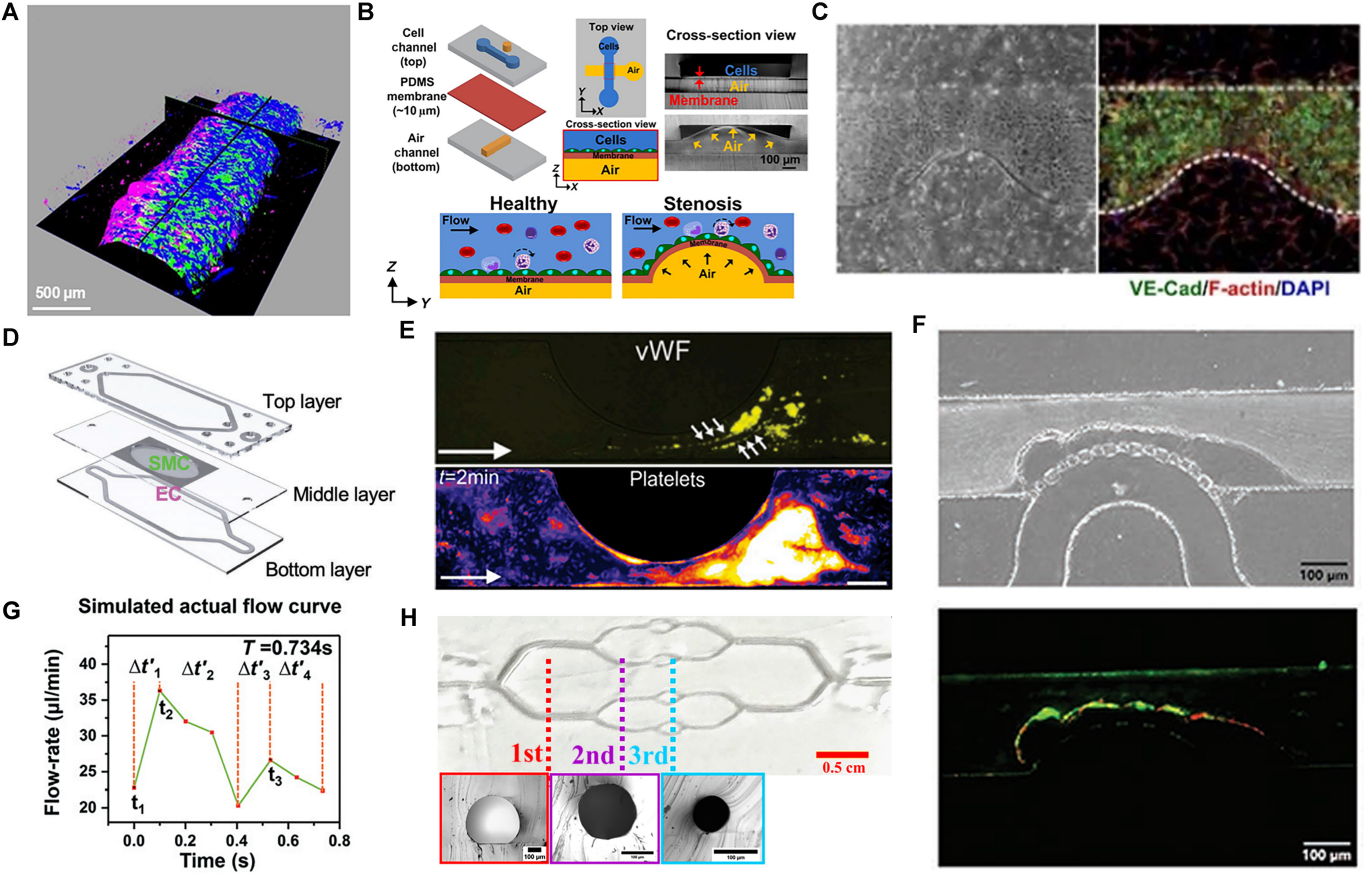
Recent advances in VoC models for AS research. (A) After 4 d of static coculture in a microfluidic system, followed by 1 d of perfusion with circulating THP-1 monocytes at a flow rate of 40 μl/min, atherosclerotic vessels were perfused with cycling THP-1 monocytes. Images reveal preservation of the vascular endothelial monolayer (green) and vascular SMC layer (blue), as well as accumulation of foam cells in the subendothelial space (red). Copyright 2024, Lippincott Williams & Wilkins. (B) Schematic illustration of an adjustable microfluidic 3D constriction model with a cell culture channel on the upper layer and an orthogonally positioned air channel on the lower layer. Copyright 2018, AIP Publishing. (C) A channel structure with a specific shape was created using collagen, featuring a deliberately designed narrow area with half the width of normal sections. Surrounding the microchannel, a layer of SMCs is closely arranged to mimic the structure of real blood vessels. Copyright 2017, Royal Society of Chemistry. (D) Coculture configuration of ECs and SMCs on both sides of a porous membrane layer in an arterial microarray mimics the vascular endothelium and facilitates intercellular material exchange. Copyright 2024, Wiley. (E) In microfluidic channels with semicircular eccentric constrictions, ECs increase the surface expression of vWF due to shear stress stimulation in the post-stenotic region. Pretreated with histamine, these ECs promote the formation of large platelet aggregates within the channel during whole blood perfusion. Copyright 2013, Proceedings of the National Academy of Sciences. (F) The hydrogel in the microfluidic chip mimics the extracellular matrix environment in blood vessels, providing a surface similar to that of real blood vessels that promotes platelet adhesion and activation. In the simulated vascular environment, platelet activation and adhesion occur upon contact with thrombogenic substances, such as collagen, on the hydrogel surface, which is the initial step in thrombus formation. Fluorescence microscopy images illustrate the binding of site-specific thrombi (green) formed with composite hydrogels to polymer nanoparticles (red) within a confined microfluidic chip. Copyright 2022, Wiley. (G) Using a digital pulse flow system to simulate blood flow rate curves and record the flow velocity within the elastic artificial vessel (EAV) through real-time monitoring, the results indicate that the flow velocity curve in the EAV exhibits a trend similar to the blood flow curve in the left coronary artery of humans. Copyright 2020, Royal Society of Chemistry. (H) By adjusting the cross-sectional dimensions of channels in microfluidic devices, researchers can simulate various sizes of blood vessels in the human microvascular system, ranging from capillaries (approximately 80 μm) to larger arteries (approximately 390 μm). Copyright 2021, ACS Publications.

Recently, researchers have investigated the use of vascular chips for point-of-care testing of cardiovascular diseases such as AS and abdominal aortic aneurysms, therefore accelerating the discovery and development of innovative treatment strategies (Fig. 4A) [40]. For instance, Menon et al. [41] developed a multilayered polydimethylsiloxane device using soft lithography. This device achieved 3D concave–convex contraction by regulating the air pressure in the bottom channel to simulate stenotic plaques (Fig. 4B). They also employed ECM patterning, based on the capillary burst valve principle, to shape the vessel geometry (Fig. 4C), enabling quantitative studies on hemodynamics and leukocyte–endothelial interactions in 3D biomimetic vascular models [42]. To mimic the intimate-media structure of blood vessels, Paloschi et al. [43] embedded porous membranes between the channels on the chip (Fig. 4D) that provided a separate growth environment for ECs and SMCs while ensuring that nutrients and signaling molecules could be freely exchanged between them.

In addition, microfluidic narrow channels provide a valuable tool for studying the molecular and cellular mechanisms of AS. A recent example using a microchannel with semicircular eccentric stenosis demonstrated that von Willebrand factor (vWF) accumulation and activity in the posterior region of the stenosis promote platelet aggregation and thrombus formation (Fig. 4E). This advancement improves the realism of blood vessel analysis models, making them more accurate simulations of AS [44]. Moreover, studies have shown that various microfluidic autosampling systems can simulate different stages of AS progression by adding specific chemical components or cytokines, or by modifying the structural and mechanical properties of the microfluidics. This capability allows for the testing of therapeutic compounds, including nanomedicines and targeted drug carriers, under conditions that better reflect the pathophysiological properties of AS (Fig. 4F) [45–49].

The progression of AS usually leads to vascular stenosis, which is commonly treated with stent implantation. However, in-stent restenosis (ISR) is a serious complication that can occur after stent implantation. To assess the potential of new stent designs, it is essential to comprehensively evaluate the effects of pulsatile blood flow, metallic stents, and anti-proliferative drugs. By adjusting the circulation flow rate and the thickness of the intermediate layer on a microfluidic chip, the fluid pressure and shear stress experienced by the cells in the deformed main channel can be modulated [50]. Lv et al. [51] developed an in vitro model of the left coronary artery by implementing a pulsatile flow system to simulate dynamic blood flow (Fig. 4G). This model demonstrates potential for personalized treatment by evaluating patient-specific conditions and optimizing treatment strategies based on unique hemodynamic profiles. Future studies should integrate computed tomography (CT) angiography image reconstruction and 3D printing technology to analyze the progression of ISR after stent implantation, using a more realistic approach.

The growing focus on the complex biophysical phenomena occurring in the small blood vessels of patients with AS has increased the demand for VoC studies, due to its capacity for high-resolution simulations of small vessel physiology and its ability to perform controlled, high-throughput experiments. Cost-efficient chips such as the design of highly ordered hierarchical microtubular networks embedded in polydimethylsiloxane (PDMS) slabs have been developed to observe the blood hemodynamic microenvironment (Fig. 4H) [52]. This technology provides crucial insights for designing of personalized treatments without the need for extensive animal or human testing. In addition, with advances in microfluidic technology, the use of printed organ-on-a-chip devices (via nozzles or light-assisted methods) and commercial platforms (e.g., OrganoPlate, Ibidi, and AIM Biotech) have become more widespread. This trend reduces the cost of 3D cell culture, making microfluidic chips more convenient and accessible for future applications [53,54].

#### Modeling organ crosstalk in AS

Recent progress in understanding the interplay between the vasculature and distant organs, especially the brain, has highlighted the critical role of disease modeling for AS, providing new insights into its systemic pathophysiology. Research has shown that neuroimmune cardiovascular interfaces (NICIs) establish an artery-to-brain circuit (ABC), which transmits inflammatory signals from arterial plaques to the central nervous system (CNS). This circuit is also influenced by sympathetic outputs that regulate local inflammatory and immune responses within the plaques [55]. The direct modulation of central neuronal activity by arterial pressure pulsations, as evidenced by synchronized local field oscillations in the olfactory bulb mitral cell layer in response to vascular pressure fluctuations, points to a rapid, intrinsic interoceptive mechanism for sensory perception modulation [56]. To delve into these interactions, vascularized brain organoid models offer a sophisticated platform for replicating brain-vascular communication in vitro. The construction of these models entails the generation of independently induced vascular and cerebral organoids from human embryonic stem cells (hESCs), followed by their fusion to produce vascularized cerebral organoids with intricate vascular networks that are closely integrated with cerebral cells [57]. This enables precise control and manipulation of the microenvironment, facilitating the study of the intricate interactions between the vasculature and the brain. The microphysiological systems can mimic the physiological and pathological conditions seen in AS, providing a platform for studying the systemic pathology of the disease. One of the key features of these models is the incorporation of extracellular vesicles (EVs), which play a crucial role in mediating intercellular signaling between the brain and vasculature [58,59]. EVs, carrying specific molecular cargo such as microRNAs, have shown potential in regulating vascular inflammation, calcification, and the progression of atherosclerotic lesions. The integration of EVs into the models allows researchers to study their role in disease progression and their potential as therapeutic targets. By integrating these cutting-edge disease models, researchers can unravel the complex organ–organ interactions underlying AS and develop innovative diagnostic and therapeutic approaches tailored to the systemic nature of the disease. The use of organs-on-chips technology provides a powerful tool for studying the interplay between the vasculature and the brain, offering a more comprehensive understanding of AS and the potential for developing personalized treatments.

## Ex Vivo AS Models

To investigate AS crosstalk and feedback mechanisms, cocultures of multiple cell types, as well as tissue and functional organ models, are essential. These models involve examining the interactions among ECs, SMCs, and macrophages, as well as the role of macrophages in lipid accumulation and inflammation, which contribute to AS progression through feedback mechanisms. Currently, ex vivo experiments in which live functional tissues or organs are isolated and incubated in an artificial environment, have been developed for these evaluations. In vitro vascular tissues are often obtained from various animal species (e.g., piglets, rabbits, or rats) due to the challenges associated with acquiring healthy human vascular tissues.

**Fig. 5.**
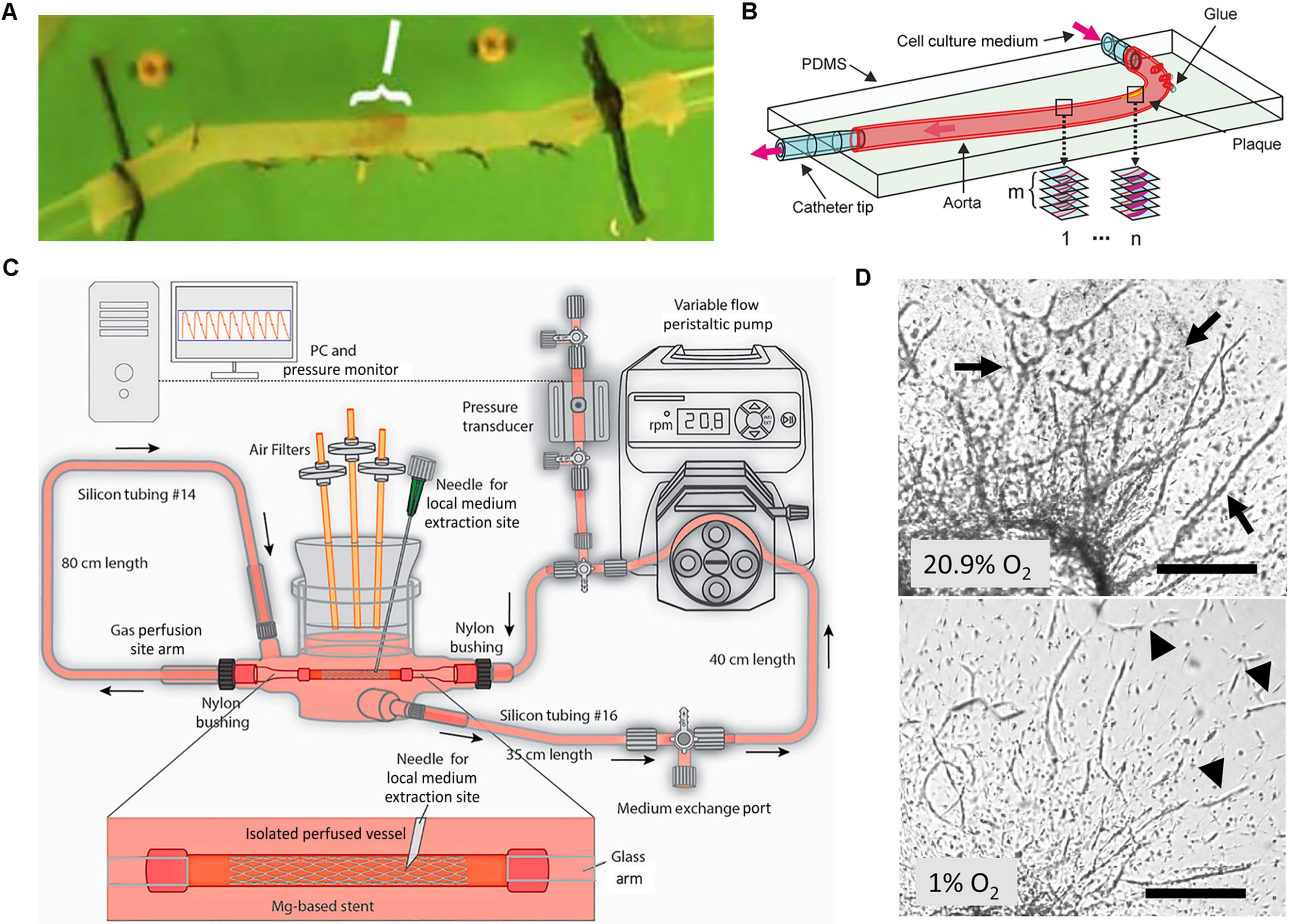
Ex vivo models for AS research. (A) Researchers administered mild compression injuries to a 3-mm section of the middle descending aorta in mice, simulating pathological processes following vascular damage ex vivo. Copyright 2010, Elsevier. (B) Mouse aortas are embedded in transparent elastic polymer matrix (PDMS) and perfused ex vivo through 2 microcatheters. Copyright 2012, SPlE Digital Library. (C) The bioreactor chamber is capable of simulating in vivo conditions, providing a culture environment for vascular samples that closely mimics physiological conditions. Copyright 2021, Elsevier. (D) Different oxygen levels significantly affect the stability and morphology of new blood vessels: Under 20.9% O_2_, structures are well-organized, while under 1% O_2_, they are fragile, tiny, and fragmented, indicating that hypoxia accelerates vascular degeneration. Copyright 2023, SpringerLink.

The combination of ex vivo experiments and computational models allow for the validation of the targeted imaging capabilities of novel fluorescent probes. In ex vivo vascular tissues, whole blood from donors can be perfused, and variables such as flow rate and pulse rate can be controlled, allowing for accurate replication of pathological microenvironments. McCarty et al. [60] induced compression injuries in isolated mouse aortas (Fig. 5A) and used contrast-enhanced ultrasound molecular imaging to detect vWF activation within the endothelium to identify high-risk AS cases. However, this system is limited in reflecting the early stages of disease progression due to the restricted access to human tissue, which is typically obtained from endarterectomy procedures targeting advanced AS [61].

Ex vivo aorta perfusion models facilitate the induction of laminar flow in catheterized arteries, maintaining the arteries in physiological conditions, and visualizing tissues without damaging the ECs. For instance, Wang et al. [62] used PDMS encapsulation of the mouse aorta to develop a perfusion model capable of generating multiple experimental endpoints, supporting long-term experiments. This model relies on controlling fluid dynamics and simulating physiological environments to maximize plaque survival (Fig. 5B). By using confocal microscopy, researchers can observe specific receptor targeting efficiency in atherosclerotic plaque cells over 24 h, with minimized motion artifacts due to polymer matrix embedding. Compared with the established in vivo experiments, ex vivo perfusion models provide enhanced control and monitoring capabilities, allowing for precise regulation of perfusion components and rapid adjustments to observe dynamic cellular responses, which ultimately leads to a substantial reduction in time, labor, and costs.

Furthermore, researchers are employing vascular bioreactors to evaluate therapies and devices under dynamic ex vivo conditions, and to investigate the fundamental mechanisms of vascular wall response. Wang et al. [63] placed porcine carotid arteries in a bioreactor system (Fig. 5C) to study and monitor mechanical conditions, such as flow and pressure. The experiments demonstrated that the apoptosis rate of the ex vivo vascular models under flow conditions was closely comparable to those of rabbit models, indicating that the in vitro model has high physiological relevance for mimicking in vivo pathological conditions. However, under static culture conditions, scaffold degradation leads to elevated Mg^2+^ concentrations and increased oxygen consumption, which in turn promote cell necrosis and apoptosis. Understanding these mechanisms is important for optimizing vascular stent therapies, particularly in the development and assessment of new absorbable vascular implants. Despite their advantages, current ex vivo vascular bioreactor models have some limitations to simulate the endothelialization process after vascular stent implantation.

Atherosclerotic plaques are commonly accompanied by pathological neovascularization and complications such as plaque progression, hemorrhage, and rupture. These conditions are typically studied using a plate-aortic arch model. To evaluate the effects of drugs on angiogenesis, vascular remodeling, and vascular diseases, the aortic ring model is employed in ex vivo research on vascular biology and drug screening. For instance, Aplin and Nicosia [64] cultured ex vivo aortic sections on matrix gel under hypoxic conditions to induce the rupture of new blood vessels (Fig. 5D). Compared with conventional in vitro tube formation models, this approach captures the entire angiogenesis process, enabling the analysis of cell proliferation, migration, blood vessel formation, microvasculature branching, perivascular recruitment, and remodeling.

Ex vivo models offer valuable insights into physiological processes and disease mechanisms by allowing controlled experimental conditions while minimizing the ethical concerns associated with clinical trials. They bridge the gap between in vitro cellular models and in vivo animal studies, providing a more accurate representation of human tissue responses. Although cellular models can measure various outcomes under controlled conditions, they often fail to account for species differences. In contrast, ex vivo models facilitate a comprehensive evaluation of AS progression, and its physicomechanical implications, thereby enhancing understanding and potentially improving therapeutic strategies.

## In Vivo AS Models

Animal models of AS are crucial preclinical tools for evaluating the efficacy and safety of new drugs and treatments in humans. The development of animal models for AS requires several key prerequisites: lipid deposition, vascular calcification, inflammation, immune system involvement, mechanical damage to the intima, and genetic modification. The methods used to create these models vary depending on factors such as the timing of AS onset, the severity of the disease, and the progression of AS lesions. Animal models of AS are essential for understanding the pathophysiology of the disease, as they can simulate the specific vascular regions most affected in human AS, namely, the coronary, cerebral, and peripheral arteries. Rodent models, such as ApoE^−/−^ and LDLR^−/−^ mice, are commonly employed in research on coronary and peripheral AS due to their ability to develop lipid-rich plaques in the aorta and peripheral vasculature. The vascular diameter and pathological characteristics of these plaques closely resemble those found in human coronary arteries, offering valuable insights into lipid metabolism and the formation of atherosclerotic plaques. Larger animal models, such as rabbits and pigs, offer more accurate representations of peripheral AS, with rabbits developing prominent lesions in the femoral and iliac arteries, while pigs, due to their similar vascular structure, are particularly valuable for studying both peripheral and coronary AS [65]. Nonhuman primates, including macaques, can develop coronary and cerebral AS, closely resembling human disease in both plaque morphology and progression, making them crucial for studying the systemic and multiterritory nature of AS [66,67]. These models, with their respective advantages and limitations, provide unique insights into the mechanisms driving AS in different vascular territories and are essential for testing new therapeutic approaches targeting AS at multiple levels.

### Diet-induced models

AS is primarily caused by lipid deposition, and the induction of a high-lipid state facilitates the development of an atherosclerotic model in arteries. Feeding animals a high-lipid diet can induce marked hyperlipidemia within weeks, and early atherosclerotic lesions may form in rabbits, pigeons, and chickens after a few months to up to 6 months, depending on the species and individual response to the diet. Rats, mice, and pigs, while challenging to maintain, can have their AS progression accelerated by incorporating eggs, bile acids, and lard to their diet [68]. In addition to high-fat and high-calorie (HFHC) diets, the inclusion of methylthioxy pyrimidine, propylthioxy pyrimidine, hypericin, and vitamin D3 can further promote lesion formation. An HFHC diet affects not only blood lipid levels but also the immune system and gut microbiome [69], all of which play important roles in AS. Moreover, given the high degree of similarity between experimental animals and humans in lipoprotein metabolism and responses to HFHC diets, future studies should analyze the correlation between total plasma cholesterol levels, lesion location, and AS characteristics. This approach will further elucidate the validity of diet-induced animal models for AS [70,71].

### Genetically engineered models

Genetic models have substantially advanced our understanding of the AS pathogenesis. Researchers have employed genetically modified mice to knock out or overexpress genes involved in lipid metabolism, inflammation, hypertension, protease activity, extracellular matrix remodeling, glucose metabolism, and immune response (Table 3). To evaluate candidate genes associated with AS, mice deficient in specific genes can be crossbred with classical transgenic models, such as LDLR^−/−^ or ApoE^−/−^ mice, and AS can be induced using an HFHC diet [72,73]. In zebrafish, gene editing is relatively straightforward, and techniques such as CRISPR/Cas9 can be employed to knock out specific genes for AS modeling. Additionally, cell type-specific promoters have been used to drive Cre expression, enabling targeted gene knockouts in specific cell types, such as ECs, macrophages, and SMCs, to investigate their roles in AS [74,75]. The use of Cre/loxP technology accelerates drug development by facilitating the rapid generation of models with diverse genetic backgrounds. As a result, transgenic animals provide faster model development, higher quality, and more accurate representation of AS progression.

**Table 3.** Innovative genetically engineered mouse models for AS research. ND, normal diet; HFD, high-fat diet; WD, Western diet.

Animal model	Diet	Limitations	Directions	References
ApoE^−/−^ mice	ND/HFD	Spontaneous hyperlipidemia and complex AS lesions	Study of lipoprotein metabolism and AS progression	[114]
LDLR^−/−^ mice	ND/HFD	Mild AS lesions without diet induction	Modeling advanced AS lesions	[115]
PCSK9-AAV mice	ND/HFD	Rapid induction of AS	Investigation of PCSK9 role in AS	[116]
ApoE3-Leiden.CETP mice	HFD	Synthetic functional ApoE; no effect on inflammation; human-like lipid profile	Study of ApoE structure–function relationship	[117]
ApoE^−/−^;Nampt-Tg mice	HFD	Exacerbates AS inflammation and promotes lesion development	Study of inflammation in AS	[118]
Cyp27a1/ApoE double knockout mice	WD	AS phenotype attenuated or absent	Study of cholesterol metabolism in AS	[119]
Ldlr-ASO mice	HFD	Inducible and reversible AS without altering lipid metabolism	Study of LDL receptor function in AS	[120]

### Accelerating the development of animal models through the induction of immune-mediated inflammation

The inflammatory process plays a critical role in the pathological changes characteristic of AS [76,77]. In particular, chronic inflammation contributes to endothelial dysfunction and plaque formation, which are the key features of AS progression. Combining an HFHC diet with an impaired immune system can accelerate the progression of AS in animal models. To further induce inflammation and mimic the immune responses involved in AS, various agents such as pathogens, bovine serum albumin (BSA), and ovalbumin (OVA) are administered to trigger an immune-mediated inflammatory response.

Pathogens commonly used in AS models include *Chlamydia pneumoniae* (CP), *Mycoplasma pneumoniae* (MP), *Helicobacter pylori*, and periodontal pathogens (*Porphyromonas gingivalis*) [78]. Among these, CP can directly infect vascular SMCs, promoting cell proliferation and migration, and ultimately contributing to the development of AS. In addition, MP infection stimulates the release of inflammatory mediators, including pro-inflammatory cytokines such as TNF-α and interleukin-1β (IL-1β), as well as chemokines like monocyte chemoattractant protein-1. These mediators attract immune cells to the damaged area, intensifying both the inflammatory response and the arterial wall damage. This process also promotes lipid deposition, further accelerating the progression of AS. In light of this, Damy et al. [79] successfully established an AS model using ApoE^−/−^ mice fed a high-cholesterol diet and concurrently administered CP and MP intraperitoneally. Vulnerable plaques characterized by increased plaque size and positive vascular remodeling, such as arterial expansion and reduced luminal narrowing, were identified in this model.

To investigate *H. pylori*-induced AS, guinea pigs are selected to serve as the primary animal model due to their similarity to humans in terms of inflammatory and immune responses to *H. pylori* infection. Plaque formation is typically observed in guinea pigs following a 60-d oral infection with *H. pylori* cultures [80]. Compared with the uninfected control group, *H. pylori* infection induces an earlier and more intense immune-inflammatory response, leading to more pronounced early AS changes observed between 8 and 12 weeks. These changes include increased expression of adhesion molecules in vascular ECs, the formation of lipid streaks, and foam cell appearance.

Furthermore, *P. gingivalis* is a major factor in periodontal disease and is used in AS models to simulate the effects of chronic infection on the vascular system, mirroring AS progression in humans. For instance, Ruan et al. [81] colonized C57BL/6 mice with *P. gingivalis* in the gingival pocket and detected accumulation of lipopolysaccharide (LPS) and gingipain in the aortic wall after 5 weeks. Consequently, *P. gingivalis* directly damages vascular ECs through its virulence factors, resulting in inflammation and dysfunction, and activates matrix metalloproteinases (MMPs), such as MMP-9, which degrade collagen and elastin within the vascular wall, potentially leading to the rupture of vulnerable plaques. Immunological approaches enable the study of AS mechanisms and offer insights into the development of anti-AS vaccines.

Moreover, the researchers have injected BSA or OVA to induce systemic immune-inflammatory responses in rabbits. When combined with a high-fat diet, this approach directly affects lipid metabolism, resulting in a more stable plaque formation process [82]. The models induced by BSA and OVA exhibit significantly thickened and fibrotic vascular walls, rendering them more suitable for studying immune-mediated vascular diseases such as giant cell arteritis and vasculitis syndromes [83]. These conditions can exacerbate the inflammatory response in AS, especially when the arterial wall is already compromised, potentially leading to further deterioration. Therefore, the injection of specific antigens to accelerate AS model development can reduce mortality rates and shorten the modeling process. Additionally, this method specifically produces vulnerable plaques, which are crucial for evaluating drug efficacy in the context of AS.

### Accelerating the development of animal models through endothelial injury

Endothelial injury is a reliable and reproducible experimental method for accelerated AS modeling, as it is a key initiator of AS. Techniques such as neural damage, endothelial drying, and balloon injury are used to induce endothelial injury in animals. When combined with high-fat diets or gene knockouts, these methods replicate a pathological process similar to human AS and significantly shorten the time required for the development of atherosclerotic lesions [84–87]. For instance, the balloon injury model can detect atherosclerotic lesions within 6 weeks, whereas the ApoE^−/−^ mouse model typically requires approximately 6 months on a high-fat diet to develop relatively mature AS. This model provides precise control over vascular intimal hyperplasia and successfully establishes lesions in the carotid artery, abdominal aorta, aortic arch, and coronary arteries, enabling researchers to observe plaque changes after various interventions with accurate plaque localization.

Furthermore, excessive iron consumption [88], guidewire induction [89], stenting [90], and photochemical exposure [91] have emerged as methods for inducing endothelial injury. These methods not only cause endothelial injury but also involve other mechanisms such as ferroptosis, thrombosis, and stent endothelial healing, all of which play substantial roles in the progression of AS.

### Accelerating the development of animal models through hemodynamic alterations

**Fig. 6.**
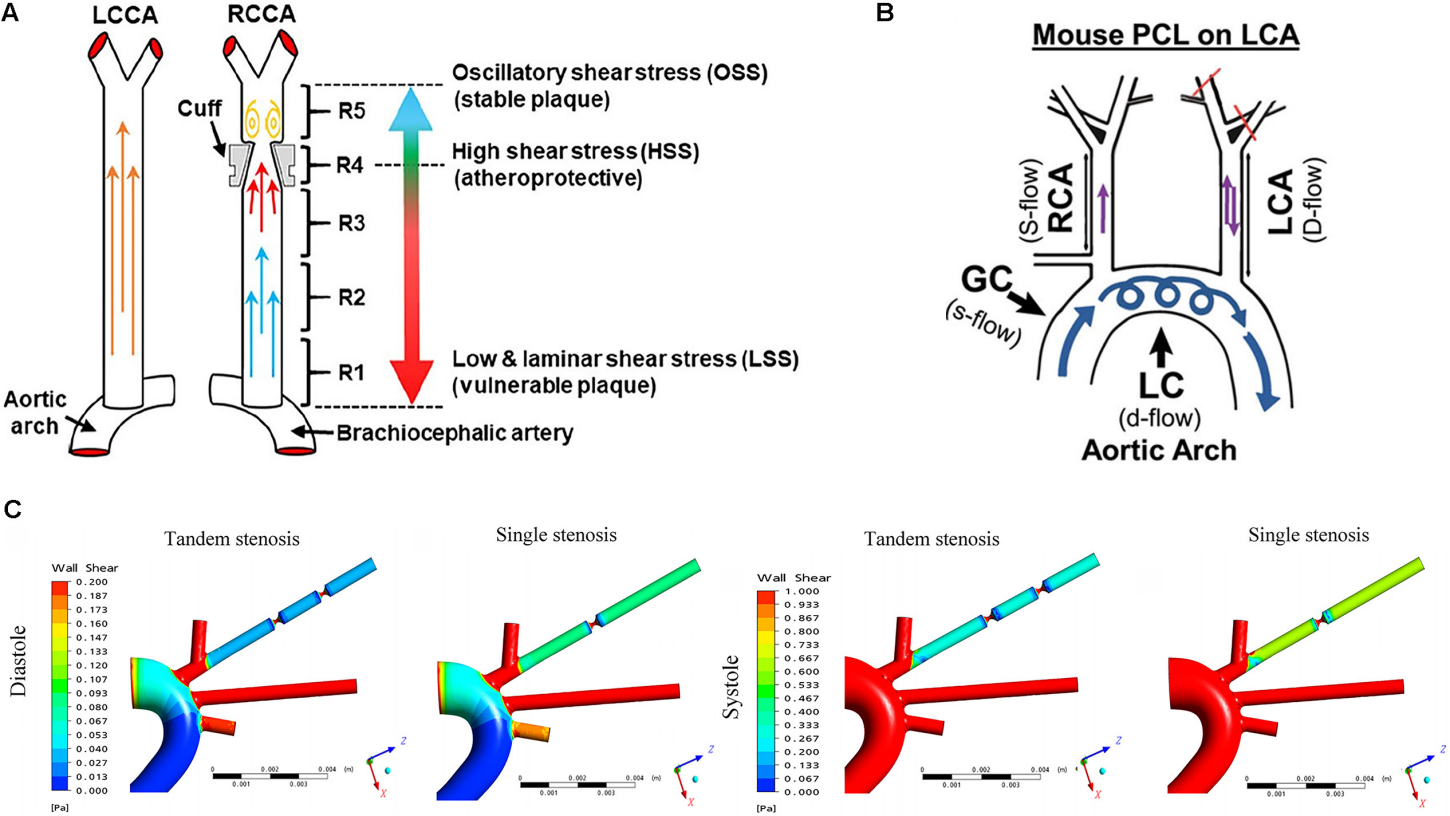
Hemodynamic alterations for accelerated AS modeling. (A) Implantation of a specially designed cuff on the RCA successfully induced plaques with varying degrees of inflammation and associated risk levels in targeted arterial regions. Copyright 2022, Springer. (B) Following PCL surgery, the right carotid artery, which maintains stable blood flow, serves as the control, whereas the ligated left carotid artery experiences disturbed blood flow. Copyright 2024, Lippincott Williams & Wilkins. (C) Computational fluid dynamics simulations of tandem and single stenosis models revealed that stenotic segments lead to reduced blood flow velocity and diminished shear stress on vascular wall. Copyright 2013, Circulation Research.

This method promotes AS formation by simulating changes in human hemodynamics, leading to functional disturbances in vascular ECs. For example, the cuff implantation model simulates stenosis by placing a silicone ring around the common carotid artery, thereby reducing its diameter. This alteration in blood flow increases flow velocity and creates regions of low shear stress downstream. This model is particularly suitable for studying the early stages of plaque formation and inflammatory responses, as these changes contribute to stable plaque formation (Fig. 6A) [92,93]. To observe the repair and remodeling processes following vascular injury, a partial carotid ligation (PCL) surgical model has been employed. Williams and colleagues [94] partially ligated the left common carotid artery (LCA), preserving the superior thyroid artery, while leaving the right common carotid artery (RCA) unligated. Following the surgery, the LCA region experiences d-flow, characterized by low velocity and fluctuating flow direction (Fig. 6B). The proliferation and migration of SMCs were observed 1 week after d-flow began to affect the vessel wall. Atherosclerotic plaques with lipid deposits and macrophage aggregates were detected in the operated area 3 weeks after PCL surgery. This approach allows researchers to evaluate the impact of therapeutic interventions on AS development within a month while simultaneously providing an opportunity for rapid screening of potential treatment options.

Additionally, studies have utilized the tandem stenosis (TS) model, in which 2 stenosis rings of different diameters are surgically implanted into mouse carotid arteries to simulate an environment with both low shear stress and high tensile stress (Fig. 6C) [95]. This setup promotes the formation of unstable plaques. Chen et al. [95] performed TS surgery on the carotid arteries of high-fat, ApoE-deficient mice. Approximately 50% of these mice exhibited characteristics similar to human vulnerable plaques within the first 7 weeks after surgery, including intraplaque hemorrhage, fibrous cap rupture, intraluminal thrombosis, and neovascularization. Thus, altering hemodynamics to create AS models is a robust research tool, characterized by its strong controllability, allowing researchers to precisely adjust parameters for model establishment and development. The study of hemodynamic characteristics in individuals has advanced the field of personalized medicine, enabling treatments to be tailored to the specific needs of patients.

## Concluding Remarks

This review discusses the advantages, current status, and key applications of biomedical models in AS. These models have provided valuable insights at both the biomolecular and cellular levels, enhancing our understanding of atherosclerotic mechanisms and facilitating the development of more effective diagnostic and therapeutic strategies for precision medicine. However, challenges persist before these models can fully achieve their therapeutic potential in AS treatment.

Given the complexity of AS progression, both the mechanisms driving its onset and the distinct pathophysiological processes at various stages, such as inflammatory response/resolution, vascular cell senescence, CHIP, and immune cell phenotypes, should be carefully considered when developing models. This will assist researchers in selecting the most appropriate models for their studies. Additionally, the interactions between platelets, immune cells, and ECs should be investigated more comprehensively in 3D AS models, particularly in the context of cardiovascular metabolism. Combining ex vivo blood vessels with 3D models can simulate complex vascular environments and hemodynamics, aiding the identification of novel therapeutic targets.

Future efforts should focus on enhancing the relevance and predictive accuracy of experimental models, ensuring that these models more accurately reflect the natural progression of human AS. To achieve this, it is essential to improve the pathophysiological fidelity of these models by incorporating advanced strategies. This includes utilizing advanced gene-editing techniques and diet/hemodynamic-induced modifications to better simulate the genetic and environmental factors that contribute to AS, allowing for more efficient modeling of disease mechanisms. Furthermore, integrating key elements such as chronic inflammation, immune cell interactions, and vascular remodeling at various stages of the disease will help capture the slow, progressive nature of AS, ensuring that models remain faithful to human pathology. Considering the growing importance of organ crosstalk in the development of AS, future construction of in vitro 3D models should not only account for the spatial microenvironment of plaques but also incorporate organ crosstalk factors, such as organoid concepts, based on research goals and lesion locations, to more accurately simulate the complex in vivo conditions. Additionally, the use of biomarker-based monitoring will enable real-time tracking of disease progression, improving model sensitivity and providing more detailed insights into plaque formation and instability. This approach allows for a more nuanced understanding of disease dynamics and enhancing the accuracy and applicability of these models for therapeutic testing. Advanced detection techniques, ranging from macroscopic to microscopic, such as optical coherence tomography, stochastic optical reconstruction microscopy, spatial transcriptomics, and single-cell genomics, will further optimize AS models for precise experimental design. By integrating imaging technologies into experimental workflows and aligning model development with emerging trends, researchers will be better equipped to bridge the gap between bench and bedside, driving innovation in the prevention and treatment of AS.
